# Dalbavancin for the Treatment of Osteomyelitis in Adult Patients: A Randomized Clinical Trial of Efficacy and Safety

**DOI:** 10.1093/ofid/ofy331

**Published:** 2018-12-10

**Authors:** Urania Rappo, Sailaja Puttagunta, Vadym Shevchenko, Alena Shevchenko, Alena Jandourek, Pedro L Gonzalez, Amy Suen, Veronica Mas Casullo, David Melnick, Rosa Miceli, Milan Kovacevic, Gertjan De Bock, Michael W Dunne

**Affiliations:** 1 Clinical Development, Allergan plc, Madison, New Jersey; 2 Orthopedic and Trauma Department, Cherkasy Regional Hospital, Cherkasy, Ukraine; 3 Medical Affairs, Allergan plc, Jersey City, New Jersey

**Keywords:** osteomyelitis, dalbavancin, gram-positive

## Abstract

**Background:**

Osteomyelitis is a challenging infection that can involve 4–6 weeks of intravenous (IV) antibiotics. Dalbavancin, approved for acute bacterial skin and skin structure infections, has potent activity against gram-positive pathogens. This study assessed the efficacy and safety of dalbavancin as a 2-dose regimen for osteomyelitis.

**Methods:**

This study was a randomized, open-label, comparator-controlled trial in adults with a first episode of osteomyelitis defined by clinical symptoms, radiologic findings, and elevated C-reactive protein. Patients were randomized 7:1 to dalbavancin (1500 mg IV on days 1 and 8) or standard of care (SOC) for osteomyelitis (oral or IV) per investigator judgment for 4–6 weeks. The primary endpoint was clinical response at day 42, defined as recovery without need for additional antibiotics in the clinically evaluable (CE) population. Clinical response was also assessed at day 21, 6 months, and 1 year.

**Results:**

Eighty patients were randomized to dalbavancin (n = 70) or SOC (n = 10). All had baseline debridement; *Staphylococcus aureus* was the most common pathogen (60% of patients). Clinical cure at day 42 was seen in 65/67 (97%) and 7/8 (88%) patients in the dalbavancin group and SOC group in the CE population, respectively. Clinical response was similar in the dalbavancin group at day 21 (94%), 6 months, and 1 year (96%). Treatment-emergent adverse events occurred in 10 patients in the dalbavancin group; no patient discontinued treatment due to an adverse event.

**Conclusions:**

A 2-dose regimen of weekly dalbavancin is effective and well tolerated for the treatment of osteomyelitis in adults.

**Clinical Trials Registration:**

NCT02685033.

Osteomyelitis in adults is a major clinical challenge and typically involves up to 6 weeks of parenteral and oral antibiotics. Debridement of infected tissue and surgical resection of necrotic bone are often needed [1, 2]. Diagnosis is established by bone biopsy with culture, in conjunction with clinical symptoms, elevated inflammatory markers such as C-reactive protein (CRP), and radiologic findings [1–3]. The most commonly isolated pathogens are *Staphylococcus aureus* and coagulase-negative staphylococci (CoNS), which can be difficult to treat [1, 3, 4]. The increasing incidence of infections caused by methicillin-resistant *S. aureus* (MRSA) and methicillin-resistant CoNS further complicates therapy, as recommended treatment includes vancomycin, which then requires an indwelling catheter, monitoring of serum drug levels, and careful dose adjustments [5–7]. Better treatment options are needed that would not require an indwelling catheter, unnecessary inpatient stays, or long-term administration of daily intravenous (IV) or oral antibiotics, in order to improve compliance and avoid associated toxicities.

Dalbavancin is a long-acting lipoglycopeptide antibiotic that inhibits cell wall synthesis, with a terminal half-life of 14.4 days [8, 9]. It is approved for the treatment of acute bacterial skin and skin structure infection (ABSSSI) as a 30-minute IV infusion in a single dose (1500 mg IV) or as 2 doses (1000 mg IV followed 1 week later by 500 mg IV) [9–13]. Dalbavancin also has potent activity against gram-positive pathogens that cause bone and joint infection, with a minimum inhibitory concentration (MIC) required to inhibit the growth of 90% of *S. aureus* isolates (MIC_90_) of 0.06 μg/mL and an MIC_99.9_ of 0.12 μg/mL, including MRSA [14, 15].

An earlier phase I study evaluated the pharmacokinetics (PK) of dalbavancin in bone and articular tissue after a 1000-mg IV infusion. Mean dalbavancin levels in bone were 6.3 μg/g at 12 hours and were sustained at 4.1 μg/g at 14 days postdose [16]. A separate phase I study evaluated the PK and safety of dalbavancin after extended-duration dosing of 1000 mg IV followed by 500 mg IV weekly for 7 additional weeks [16]. Population PK modeling from these 2 studies led to a proposed dosing regimen of two 1500-mg IV infusions 1 week apart to provide tissue exposure above the dalbavancin MIC_99.9_ of 0.12 μg/mL for *S. aureus* for 8 weeks, with a similar overall area under the curve (AUC) as a regimen of 1000 mg IV followed by 4 weekly doses of 500 mg IV [16].

The objective of this clinical trial was to describe the efficacy and safety of dalbavancin for the first episode of osteomyelitis known or suspected to be caused by gram-positive pathogens in adults.

## METHODS

### Study Conduct

This study was a phase II, single-center, randomized, open-label, comparator-controlled, parallel-group study conducted between March 2016 and December 2017 in Cherkasy Regional Hospital, an 860-bed tertiary care teaching hospital in Cherkasy, Ukraine. The site had participated in the 3 pivotal dalbavancin ABSSSI trials, and as a large orthopedic referral center for 20 regions, it could complete enrollment in a reasonable time frame with an efficient collection of reliable data, including bone cultures. The protocol was approved by the independent ethics committee and government agencies of the Ukraine (approval No. 778) and by the European Union Clinical Trial Directive (Directive 2001/20/EC). The study was conducted in accordance with the Declaration of Helsinki. All patients provided written informed consent.

### Study Patients

Patients enrolled were ≥18 years old with a first episode of osteomyelitis with (1) pain or point tenderness upon palpation or probing to bone, (2) radiological findings (plain radiograph or magnetic resonance imaging [MRI]) consistent with osteomyelitis, and (3) elevated CRP above the upper limit of normal. Key exclusion criteria included >24 hours of IV antibiotics within 96 hours of randomization, prosthetic material at the site of infection at study initiation, a prior episode of osteomyelitis or a failed course of therapy for osteomyelitis, infection associated with a burn wound, sacral decubitus ulcer, multiple sites of osteomyelitis, septic arthritis that was noncontiguous to osteomyelitis, gram-negative bacteremia, or concomitant endocarditis or necrotizing fasciitis. Patients with acute or chronic osteomyelitis were enrolled if they met inclusion criteria.

### Randomization and Intervention

Patients were randomized 7:1 to dalbavancin 1500 mg IV as a 30-minute infusion on day 1 and day 8 or to standard of care (SOC) for osteomyelitis (oral or IV) based on investigator judgment for 4–6 weeks (Supplementary Figure 1). A list of patient randomization numbers was generated by the sponsor statistical programmer and was provided to the site with corresponding treatment group assignment, which was concealed with scratch-off labels that were opened just before randomization, to minimize patient selection bias. Randomization numbers were assigned consecutively by the investigator at the time each patient was randomized. The dalbavancin dose was adjusted to 1000 mg IV by the pharmacist or designee for patients not on dialysis with a creatinine clearance of <30 mL/minute. Aztreonam was allowed at randomization for presumed gram-negative coinfection and could be discontinued if a gram-negative pathogen was not identified; a switch to an oral antibiotic for gram-negative coverage was allowed after clinical improvement only for those with a gram-negative coinfection identified at baseline. Patients with gram-negative pathogens only in baseline bone cultures were discontinued from study drug per protocol, received appropriate antibiotics for gram-negative infections, and remained in the study for follow-up.

### Endpoints

The primary endpoint was clinical response at day 42 in the clinically evaluable (CE) population, defined as recovery without need for additional antibiotic therapy. Failure was defined as any of the following: additional antibiotics for no response or worsening after improvement, new purulence, amputation due to progression of infection, >6 weeks of antibiotics in the comparator arm, or death (for any reason). Indeterminate status was defined as loss to follow-up or amputation due to vascular insufficiency. Clinical response was also assessed at day 180 (6 months) and day 365 (1 year); early clinical improvement was assessed at day 21 as no worsening of pain and/or point tenderness relative to baseline and again at day 28, now also associated with a decrease in CRP.

The modified intent-to-treat (mITT) population includes patients who received any amount of randomized medication and met criteria for known or suspected gram-positive osteomyelitis; it excludes patients with only a gram-negative pathogen isolated from blood and/or bone culture. The CE population is a subset of the mITT population that also received ≥1 dose of dalbavancin (or ≥2 weeks of SOC) as randomized, and no more than 1 dose of a non-study systemic antibiotic with activity against the causative organism for an indication other than osteomyelitis. The microbiological-mITT (micro-mITT) population is a subset of the mITT population that had a gram-positive pathogen isolated from blood and/or bone. The safety population includes all randomized patients who received any amount of randomized medication.

Safety data were collected at each visit (baseline, days 1, 8, 21, 28, 42, 6 months, and 1 year) and included adverse events, physical examination, chemistry and hematology assessments (baseline, days 8 and 28), erythrocyte sedimentation rate (ESR) and CRP (baseline, days 8, 28, 42, and 6 months), and imaging with plain film or MRI (baseline, days 28, 42, and 6 months).

### Statistical Analysis

Statistical Analysis Software (SAS), version 9.3, was used for statistical analyses. Descriptive statistics, including the numbers and percentages for categorical variables and the numbers, means, standard deviations, medians, minimums, and maximums for continuous variables, were provided. *P* values were obtained using the Fisher exact test for the analysis of categorical variables and Wilcoxon rank-sum test for the analysis of continuous variables. Two-sided 95% confidence intervals (CIs) for the percentage of patients with clinical cure were obtained using the Clopper-Pearson method. Safety data were coded using version 20.1 of the Medical Dictionary for Regulatory Activities (MedDRA Maintenance and Support Services Organization).

## RESULTS

### Patients

**Figure 1.**
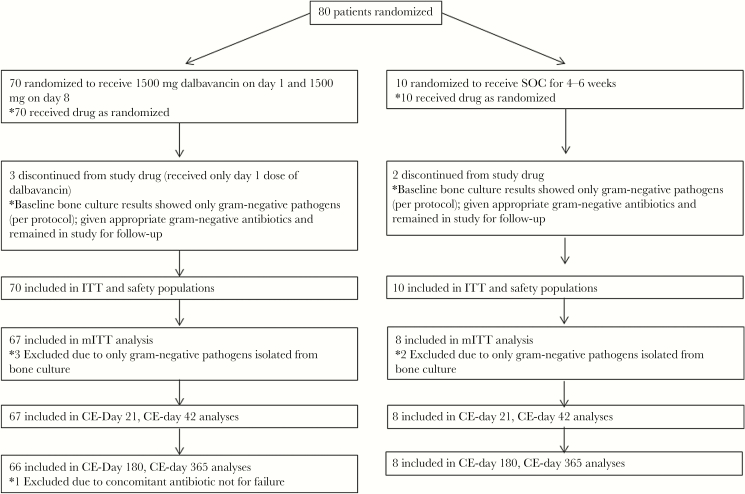
Patient disposition. Abbreviations: CE, clinically evaluable; ITT, intent-to-treat; micro-mITT, microbiological mITT; mITT, modified intent-to-treat; SOC, standard of care.

A total of 81 patients were assessed for eligibility; 1 was a screen failure due to not meeting inclusion criteria. Eighty patients were randomized to dalbavancin (n = 70) or SOC (n = 10), as specified in the study protocol; all received study drug, and the trial ended after the last enrolled patient completed the study (Figure 1). Patients on SOC received an IV comparator twice daily for 4–6 weeks (Supplementary Table 1); the most common regimens were vancomycin IV for 29–30 days (n = 3) or vancomycin IV for 5–16 days with switch to linezolid IV or levofloxacin IV to complete 29 days of therapy (n = 4). The mITT and CE populations at day 42 included 67/70 (96%) dalbavancin patients and 8/10 (80%) SOC patients. Three patients in the dalbavancin arm received only the day 1 dalbavancin dose, and 2 patients in the SOC arm discontinued study drug per protocol; the reason for the premature discontinuation from study drug in both arms was that only gram-negative pathogens were isolated from bone at baseline, and this was also the reason that they were excluded from the mITT population (Figure 1). All other patients in the dalbavancin group completed both doses of dalbavancin.

Demographics and baseline disease characteristics of each treatment group are shown in Table 1. Ten patients (14.3%) in the dalbavancin group and 5 (50%) in the SOC group had a history of diabetes mellitus, including 4 patients and 1 patient with baseline diabetic foot infections, respectively. There was a higher percentage of men in the dalbavancin group (*P* = .024) and a higher percentage of patients with diabetes in the SOC group (*P* = .017); no other differences were statistically significant. The most common sites of osteomyelitis were the tibia, foot, and femur. Mean baseline CRP was 43.9 mg/L and 20.4 mg/L in the dalbavancin and SOC groups, respectively. All patients had baseline debridement and open biopsy with culture; bone histology showed acute inflammatory cells in 80% of patients and necrotic bone consistent with chronic osteomyelitis in 61.4% of dalbavancin patients and 60% of SOC patients. A pathogen was isolated from bone in 65/70 (93%) patients in the dalbavancin group and 10/10 (100%) patients in the SOC group; *S. aureus* was the most common pathogen. Four patients in the dalbavancin group (2 with MSSA and 2 with CoNS) and 1 in the SOC group (CoNS) had bacteremia at baseline. No patient developed an organism that was not susceptible to dalbavancin during the study. Concomitant aztreonam was used in 11.4% of dalbavancin patients and 10% of SOC patients; none of the patients in either group received follow-up oral gram-negative coverage.

**Table 1.** Demographics and Baseline Patient and Disease Characteristics in the Safety Population

Characteristic^a^	Dalbavancin (n = 70)	Standard of Care (n = 10)
Age, mean (SD; range), y	49.2 (13.3; 26–79)	54.4 (15.3; 29–79)
Male sex^b^	59 (84.3)	5 (50.0)
Race		
White	70 (100)	10 (100)
Ethnicity		
Not Hispanic/Latino	70 (100)	10 (100)
Body mass index, kg/m^2^		
Mean (SD)	26.1 (5.1)	30.7 (7.4)
Median (min, max)	24.7 (18.6, 40.1)	33.8 (21.6, 40.3)
Diabetes^c^	10 (14.3)	5 (50.0)
Predisposing factors at site of osteomyelitis		
Prior fracture and surgical repair	33 (47.1)	4 (40.0)
Prior fracture	2 (2.9)	0
Surgical intervention		
Debridement and open biopsy	70 (100)	10 (100)
Vacuum-assisted closure of wound	8 (11.4)	3 (30)
Skin graft	1 (1.4)	1 (10)
Aztreonam use	8 (11.4)	1 (10)
Site of osteomyelitis^d^	Tibia: 27 (38.6) Foot: 17 (24.3) Femur: 11 (15.7) Humerus: 4 (5.7) Hand: 4 (5.7) Ulna: 1 (1.4) Fibula: 2 (2.9) Pelvic bone: 1 (1.4) Other: 3 (4.3)	Tibia: 2 (20) Foot: 2 (20) Femur: 4 (40) Fibula: 1 (10) Pelvic bone: 1 (10)
Baseline diabetic foot infection	4 (5.7)	1 (10)
Baseline CRP, mg/L^e^		
Mean	43.9	20.4
Median	24	18
Baseline ESR, mm/h^f^		
Mean	33.2	30.6
Median	34	24
Baseline bacteremia		
MSSA	2 (3)	0
Coagulase-negative staphylococci	2 (3)	1 (10)
Baseline pathogen in bone^g^	65 (92.9)	10 (100)
* Staphylococcus aureus*	42 (60)	6 (60)
MSSA	38 (54.3)	5 (50)
MRSA	4 (5.7)	1 (10)
Coagulase-negative staphylococci		
* Staphylococcus epidermidis*	6 (8.6)	2 (20)
* Staphylococcus haemolyticus*	4 (5.7)	0
* Staphylococcus hominis*	2 (2.9)	0
* Staphylococcus pasteuri*	1 (1.4)	0
* Staphylococcus simulans*	1 (1.4)	0
Enterococci		
* Enterococcus faecalis*	7 (10)	1 (10)
* Enterococcus faecium*	1 (1.4)	0
Anaerobes	9 (12.9)	0
Streptococci		
* Streptococcus agalactiae*	1 (1.4)	1 (10)
* Streptococcus dysgalactiae*	1 (1.4)	0
* Streptococcus pyogenes*	1 (1.4)	0
Other gram-positive pathogens		
* Corynebacterium striatum*	2 (2.9)	1 (10)
*Aerococcus viridans*	1 (1.4)	0
* Globicatella* species	1 (1.4)	0
* Micrococcus luteus*	1 (1.4)	0
Mixed (gram-positives and aerobic gram-negatives)	11 (15.7)	2 (20)
Gram-negative pathogens only^h^	3 (4.3)	2 (20)
No growth^i^	5 (7.1)	0
Baseline bone histology		
Acute inflammatory cells	56 (80)	8 (80)
Necrotic bone	43 (61.4)	6 (60)
Edema	11 (15.7)	4 (40)
Granulations	8 (11.4)	1 (10)
Vascular congestion	4 (5.7)	0

Abbreviations: CRP, C-reactive protein; ESR, erythrocyte sedimentation rate; ITT, intent-to-treat; MRSA, methicillin-resistant *Staphylococcus aureus*; MSSA, methicillin-susceptible *S. aureus*; SOC, standard of care.

^a^Data are presented as No. (%) unless otherwise specified.

^b^ *P* = .024.

^c^ *P* = .017.

^d^“Other” sites include patella (n = 1), clavicle (n = 1), finger (n = 1).

^e^CRP normal range = 0–6 mg/L.

^f^ESR normal range = 1–10 mm/h.

^g^Categories are not mutually exclusive.

^h^Three patients in the dalbavancin arm and 2 patients in the SOC arm were premature discontinuations from study drug due to only gram-negative pathogens isolated from bone culture.

^i^Five patients in the dalbavancin arm had no growth on bone biopsy; histology results showed necrotic bone in 3/5 (60%) and acute inflammatory cells in 2/5 (40%).

### Efficacy Outcomes

**Figure 2.**
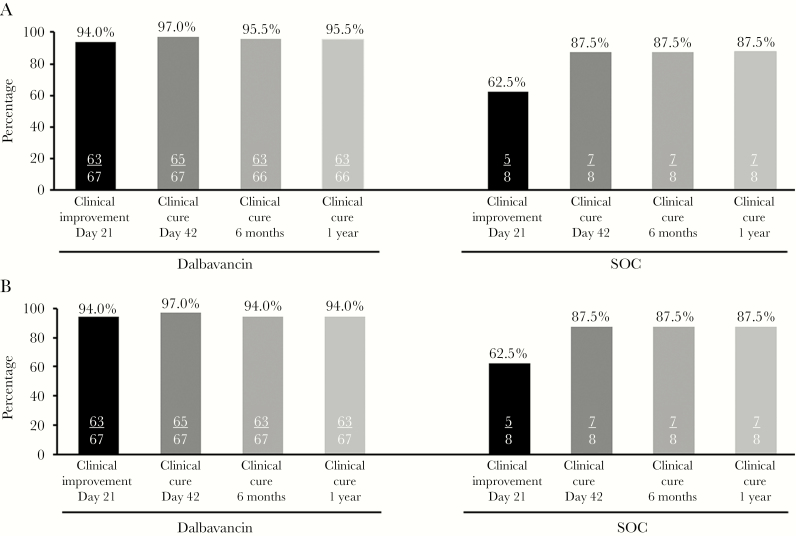
Clinical outcomes in the (A) CE and (B) mITT populations. Abbreviations: CE, clinically evaluable; CRP, C-reactive protein; IV, intravenous; mITT, modified intent-to-treat; SOC, standard of care.

Clinical cure at day 42 in the CE and mITT populations was seen in 65/67 (97%) patients in the dalbavancin group and 7/8 (88%) patients in the SOC group (Figure 2). There were no failures in the dalbavancin group at day 42; 2 patients were indeterminate due to loss to follow-up before day 21, and both had clinical improvement with decreased pain and point tenderness on day 8. There was 1 failure in the SOC group at day 42, due to having received >6 weeks of antibiotic therapy; he received 7 weeks of SOC antibiotics based on investigator judgment and clinical status. The 95% CI for the primary endpoint of clinical cure at day 42 in the CE population was 89.6–99.6 in the dalbavancin group and 47.3–99.7 in the SOC group. Clinical cure at 6 months and 1 year was seen in the dalbavancin group in 63/66 (95%) of the CE and 63/67 (94%) of the mITT populations; clinical cure was seen in 7/8 (88%) patients in the SOC group in both populations. Four patients in the dalbavancin group did not meet criteria for clinical cure in the mITT population at 6 months and 1 year: 2 were indeterminate due to loss to follow-up, 1 was a clinical failure due to death from an underlying medical condition approximately 5 months after the study drug, unrelated to treatment with dalbavancin, and 1 was a clinical failure due to requiring additional antibiotics approximately 4 months after the study drug for soft tissue inflammation at the site of prior bone biopsy; he had debridement of soft tissue with no signs of inflammation in the bone and no growth from repeat site cultures. One patient in the SOC group did not meet criteria for clinical cure in the mITT population at 6 months and 1 year due to indeterminate status from loss to follow-up before the 6-month visit; this patient was categorized as a clinical cure at the day 42 visit.

Clinical improvement at day 21 in the mITT and CE populations was seen in 63/67 (94%) patients in the dalbavancin group, and in 5/8 (63%) patients in the SOC group; an additional analysis of improvement at day 28 (no worsening of pain and/or point tenderness relative to baseline) with decrease in CRP measured at day 28 yielded identical results. The reasons for not meeting criteria for clinical improvement at day 21 were: in the dalbavancin group, 2 patients had missing CRP values due to loss to follow-up and 2 had increased CRP; in the SOC group, 2 patients had no change in CRP and 1 had increased CRP.

All (4/4) patients with baseline gram-positive bacteremia in the micro-mITT population were in the dalbavancin group, had the same pathogens isolated from bone as from blood (2 with MSSA and 2 with CoNS), and cleared their bacteremia by the second set of blood cultures, drawn 2–3 days later, with no recurrence of bacteremia. All patients with diabetic foot infection in the dalbavancin group (4/4) and the SOC group (1/1) were clinical cures at day 42, 6 months, and 1 year.

In the micro-mITT population, 60/62 (96.8%) patients in the dalbavancin group and 7/8 (87.5%) patients in the SOC group were clinical cures at day 42, with no failures in the dalbavancin group at day 42. Clinical cure by baseline pathogen is shown in Supplementary Table 2. All baseline gram-positive isolates from bone or blood cultures were susceptible to dalbavancin, with dalbavancin MICs spanning a narrow range: 0.06 to 0.12 μg/mL for *S. aureus* and 0.06 μg/mL for *Enterococcus faecalis* and *Enterococcus faecium*. The highest dalbavancin MIC for any gram-positive isolate was 0.25 μg/mL in 2 patients in the dalbavancin group: *Staphylococcus haemolyticus* (n = 1) and *Corynebacterium striatum* (n = 1). All gram-positive pathogens in the micro-mITT population were susceptible to vancomycin (MIC ≤ 2 μg/mL), with a vancomycin MIC of 2 μg/mL for the following isolates: *Staphylococcus epidermidis* (n = 9), *E. faecalis* (n = 4), MSSA (n = 2), and *S. haemolyticus* (n = 1).

In the dalbavancin group, all 11 patients with mixed infections in bone (Supplementary Table 3) were clinical cures at all timepoints; 3 received adjunctive gram-negative coverage with aztreonam for an average (range) of 8.7 (6–12) days. All 3 patients in the dalbavancin group with gram-negative infections only in bone were discontinued from the study drug per protocol, received gram-negative antibiotics, and were clinical cures at all timepoints. In the SOC group, 2 patients had mixed infections in bone (Supplementary Table 3): 1 received an antibiotic with gram-negative coverage and was considered a clinical failure at day 42 (due to >6 weeks of antibiotics) and clinical cure at 6 months and 1 year; 1 received an antibiotic with gram-negative coverage and was a clinical cure at all timepoints. Of 2 patients in the SOC group with gram-negative infections only in bone, 1 received a gram-negative antibiotic and was indeterminate at all timepoints (due to withdrawal/loss to follow-up); 1 received a gram-negative antibiotic and was a clinical cure at all timepoints.

The mean length of hospital stay was 15.8 days in the dalbavancin group and 33.3 days in the SOC group (*P* < .001) (Table 2). Patients in the dalbavancin group received a 30-minute infusion on days 1 and 8 for a total IV duration of 1 hour; patients in the SOC group received an average of 31.6 days of twice-daily IV antibiotic therapy for an average total IV duration of 101.3 hours per patient.

**Table 2.** Hospital Stay and Antibiotic Treatment (mITT Population)

Outcome	Dalbavancin (n = 67)	Standard of Care (n = 8)
Length of hospital stay, d		
Mean ± SD	15.8 ± 7.1	33.3 ± 14.2
Median (range)	15.0 (8–38)	30.5 (11–56)
Days of IV antibiotic treatment		
Mean ± SD	2.0 ± 0	31.6 ± 7.0
Median (range)	2 (2–2)	29 (29–49)
Total IV infusion duration, h		
Mean ± SD	1.0 ± 0.02	101.3 ± 20.8
Median (range)	1.0 (1.0–1.1)	112.6 (66.9–113.3)

Abbreviations: IV, intravenous; mITT, modified intent-to-treat.

CRP decreased from baseline to 6-month follow-up in both groups (Supplementary Figure 2). All patients had elevated CRPs at baseline; the mean CRP was 42.1 mg/L in the dalbavancin group and 22.5 mg/L in the SOC group at baseline. Patients in the dalbavancin group with the highest baseline CRPs (192 mg/L) had the following pathogens in bone: MSSA (n = 4), MRSA (n = 1), and mixed gram-positive and gram-negative pathogens (n = 1). CRP decreased in all these patients by day 28, including 4 with normal CRP (6 mg/L); CRP was normal (6 mg/L) in all patients in the study at 6 months (n = 5). All 6 patients were clinical cures at day 42, as were all patients in the study at 6 months and 1 year (n = 5).

### Safety

Treatment-emergent adverse events (TEAEs) were reported in 10 (14.3%) patients in the dalbavancin group and in no patients in the SOC group (Table 3). The most common TEAEs, each reported in 2 patients in the dalbavancin group, were anemia (associated with baseline debridement) and impaired healing, assessed as not related to the study drug. Two patients had serious TEAEs approximately 4–5 months after the study drug that were assessed as not related to the study drug. There were no patients who discontinued the study drug due to TEAEs. There were no observed safety concerns in clinical laboratory parameters.

**Table 3.** Patients With Treatment-Emergent Adverse Events (Safety Population)

Adverse Event	Dalbavancin, No. (%) (n = 70)	SOC, No. (%) (n = 10)
Patients experiencing ≥1 of:		
TEAE	10 (14.3)^a^	0 (0)
Drug-related TEAE	1 (1.4)	0 (0)
Serious TEAE	2 (2.9)^b^	0 (0)
Death	1 (1.4)^c^	0 (0)
TEAE leading to premature discontinuation of study drug	0 (0)	0 (0)

Abbreviation: TEAE, treatment-emergent adverse event.

^a^Includes 3 patients with onset of TEAEs after day 42 (primary endpoint), not related to the study drug.

^b^Both serious TEAEs were not related to the study drug and occurred after day 42.

^c^Due to heart failure approximately 5 months after the last dalbavancin dose.

## DISCUSSION

The clinical trial presented here represents one of the largest randomized comparative clinical trials in osteomyelitis [17]. Its purpose was to describe the efficacy and safety of dalbavancin for the treatment of osteomyelitis known or suspected to be caused by gram-positive pathogens in adults. The majority of patients in this trial had a gram-positive organism isolated from bone, consistent with previous literature. The dalbavancin group had high clinical cure rates at day 42 (97% in the CE, mITT, and micro-mITT populations), sustained through 1 year. The 95% CI for the primary endpoint of clinical cure at day 42 had a lower limit of approximately 90% in the dalbavancin group, suggesting that the data from this group alone are good evidence of a treatment effect, especially given the lack of an approved drug for osteomyelitis to use as a comparator. These findings are consistent with the efficacy of dalbavancin in an animal model with MRSA sternal osteomyelitis [18] and recent anecdotal observations of high response rates in the treatment of osteomyelitis with dalbavancin, associated with a reduced length of stay and treatment costs [19, 20].

Dalbavancin was well tolerated in this study; no patient discontinued treatment due to an adverse event, and no serious adverse events were considered related to dalbavancin. All patients had a baseline bone culture, with 89% and 80% of patients with a gram-positive pathogen in the dalbavancin and SOC groups, respectively. Most patients achieved clinical cure regardless of the baseline pathogen. Despite the low rate of gram-negative therapy in patients in the dalbavancin group (3/11) with concomitant gram-negative aerobes in bone, all 11 patients with mixed infections were sustained clinical cures at all timepoints, suggesting that the gram-positive organisms in these patients were the most clinically important.

Recently, there has been increased interest in oral antibiotics as SOC for treatment of osteomyelitis [21, 22]. Although oral antibiotics do not require an indwelling catheter, their prolonged use is associated with increased risk of poor adherence, gastrointestinal intolerance, and inconsistent serum levels [21]. Dalbavancin may provide a simplified, 2-dose, alternative treatment option, ensuring 100% adherence and minimal impact on gastrointestinal flora [23].

Patients in the dalbavancin group had a total IV duration of 1 hour, whereas patients in the SOC group had an average total IV duration of 101.3 hours per patient. The brief 2-dose dalbavancin regimen may be suitable for an outpatient setting, such as emergency rooms, infusion centers, and hospital outpatient departments [24].

Limitations of this study include the open-label, single-center design in Ukraine and the small sample size in the SOC group. An open-label design was chosen due to challenges with conducting a blinded study with various treatment options and dosing schedules. Although the focus of the trial was on obtaining as much data as possible on dalbavancin outcomes, benchmarking the outcomes against the local standard of care in a small subset of patients highlighted the unique characteristics of this 2-dose regimen. A small SOC group was preferred over extrapolating from historical controls that may differ from the study cohort and would lack efficacy data and inflammatory markers at the same timepoints as the dalbavancin group. The length of stay (LOS) was significantly shorter in the dalbavancin group than in the SOC group, though if the study was conducted in the United States, the LOS may have been shorter in both study groups. Another limitation is that native osteomyelitis was the objective of this trial; these results could serve as the basis for further study of osteomyelitis after joint replacement rather than direct extrapolation of these data.

There are significant potential advantages in a 2-dose regimen of dalbavancin providing 6 weeks of antibiotic coverage for the treatment of osteomyelitis. The high microbiologic potency of dalbavancin combined with the prolonged tissue exposure at the site of infection and high clinical cure rates observed in this study make dalbavancin a convenient and effective option for an infection that has historically been burdensome for patients, providers, and the health care system.

## Supplementary Data

Supplementary materials are available at *Open Forum Infectious Diseases* online. Consisting of data provided by the authors to benefit the reader, the posted materials are not copyedited and are the sole responsibility of the authors, so questions or comments should be addressed to the corresponding author.

ofy331_suppl_supplementary_material

## Notes

Prior presentations: Oral session 1172, ECCMID; April 22–25, 2017; Vienna, Austria. Poster 105, ASM/ESCMID Conference on Drug Development to Meet the Challenge of Antimicrobial Resistance; September 6–8, 2017; Boston, Massachusetts. Mini-oral ePoster session O0697, ECCMID; April 21–24, 2018; Madrid, Spain.
