# Supplementary material for: Dalbavancin for the Treatment of Osteomyelitis in Adult Patients: A Randomized Clinical Trial of Efficacy and Safety
Source: Open Forum Infect Dis. 2018 Dec 10;6(1):ofy331. doi: 10.1093/ofid/ofy331 (PMC6326511; doi:10.1093/ofid/ofy331)

Supplementary Material

Supplementary Figure 1. Study design. EOT=end of treatment; IV=intravenous.

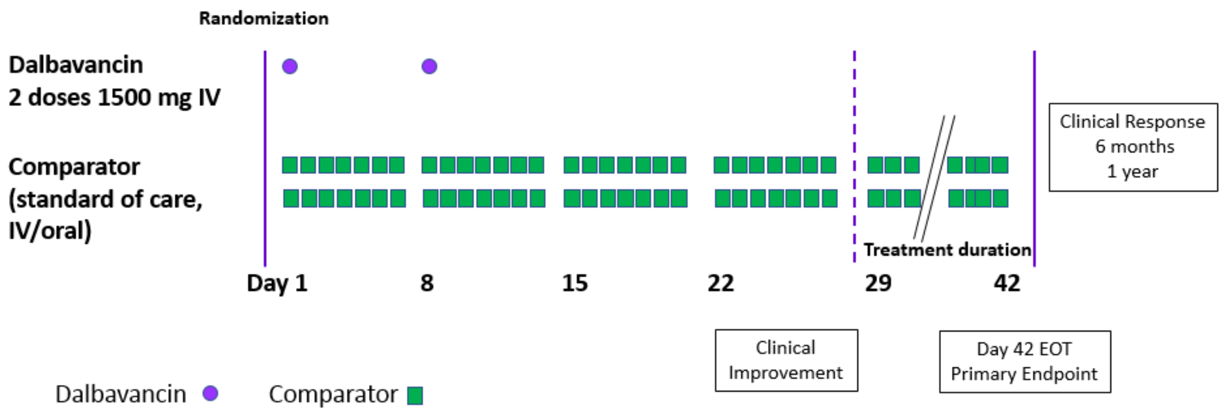

Supplementary Table 1. All Treatment Regimens for Patients Receiving Standard of Care (Safety Population)

Regimen	SOC, no./No. (%)	Baseline Pathogen(s) in Bone
Vancomycin IV x 29–30 d (D1–29 or D1–30)	3/10 (30)	*Corynebacterium striatum* (n=1); *Staphylococcus epidermidis* (n=1); MSSA + *Enterococcus faecalis* (n=1)[a]
Vancomycin IV x 5 d (D1–5); linezolid IV x 25 d (D5–29)	1/10 (10)	MSSA + *Staphylococcus epidermidis* + *Streptococcus agalactiae*
Vancomycin IV x 6 d (D1–6); linezolid IV x 24 d (D6–29)[b]	1/10 (10)	MRSA + *Klebsiella pneumoniae* + *Proteus mirabilis*
Vancomycin IV x 8 d (D1–8); levofloxacin IV x 22 d (D8–29)	1/10 (10)	MSSA
Vancomycin IV x 16 d (D1–16); levofloxacin IV x 15 d (D15–29)	1/10 (10)	MSSA
Vancomycin IV × 7 d (D1–7); linezolid IV plus cefotaxime IV x 43 d (D7–49)[c]	1/10 (10)	MSSA + *Pseudomonas aeruginosa* + *Raoultella planticola* + *Serratia marcescens*
**Excluded from mITT population due to only Gram-negative pathogens in bone**		
Vancomycin IV x 16 d (D1–16); amikacin IV x 13 d (D8–20)[d,e]	1/10 (10)	*Klebsiella pneumoniae* + *Proteus mirabilis*

Vancomycin IV x 5 d (D1–5); IV ceftriaxone x 25 d (D4–29)[d]	1/10 (10)	*Enterobacter cloacae complex* + *Escherichia coli* + *Klebsiella oxytoca*

IV=intravenous; all regimens were q12h. d=days; D=Study Day. MRSA=methicillin-resistant *Staphylococcus aureus*; MSSA=methicillin-susceptible *S. aureus*; SOC=standard of care; mITT=modified intent-to-treat

[a]Indeterminate at 6 months and 1 year due to loss to followup. [b]Received adjunctive aztreonam. [c]Clinical failure at day 42 due to receipt of antibiotics > 6 weeks. [d]Discontinued study drug per protocol when baseline bone culture results showed only gram-negative pathogens; given appropriate gram-negative antibiotic. [e]Withdrew from study on day 20 (withdrew consent)

Supplementary Table 2. Clinical Cure at Day 42 by Baseline Pathogen (CE Population)

Pathogen, n/N (%)[a]	Dalbavancin	Standard of Care
**Gram-positive aerobes**		
Staphylococcus aureus	41/42 (97.6)[b]	5/6 (83.3)[c]
MRSA	4/4 (100)	1/1 (100)
MSSA	37/38 (97.4)[b]	4/5 (80.0)[c]
Coagulase-negative staphylococci	12/14 (85.7)[d]	2/2 (100)[e]
Enterococcus faecalis	7/7 (100)	1/1 (100)
Enterococcus faecium	1/1 (100)	0
Streptococcus agalactiae	1/1 (100)	1/1 (100)
Streptococcus dysgalactiae	1/1 (100)	0
Streptococcus pyogenes	1/1 (100)	0
Corynebacterium striatum	2/2 (100)	1/1 (100)
Aerococcus viridans	1/1 (100)	0
Globicatella spp	1/1 (100)	0
Micrococcus luteus	1/1 (100)	0
**Gram-positive anaerobes**		
Peptoniphilus harei	2/2 (100)	0
Anaerococcus prevotii	1/1 (100)	0
Finegoldia magna	1/1 (100)	0
Peptostreptococcus anaerobius	1/1 (100)	0
**Gram-negative aerobes**		
Escherichia coli	3/3 (100)	0

Klebsiella pneumoniae	2/2 (100)	1/1 (100)
Enterobacter cloacae complex	2/2 (100)	0
Morganella morganii	2/2 (100)	0
Pseudomonas aeruginosa	2/2 (100)	0/1 (0)[c]
Proteus mirabilis	1/1 (100)	1/1 (100)
Raoultella planticola	1/1 (100)	0/1 (0)[c]
Acinetobacter calcoaceticus	1/1 (100)	0
Escherichia hermannii	1/1 (100)	0
Pluralibacter gergoviae	1/1 (100)	0
Serratia marcescens	0	0/1 (0)[c]
**Gram-negative anaerobes**		
Bacteroides fragilis	2/2 (100)	0
Bacteroides thetaiotaomicron	1/1 (100)	0
Bacteroides vulgatus	1/1 (100)	0
Porphyromonas asaccharolytica	2/2 (100)	0
Prevotella melaninogenica	2/2 (100)	0
Prevotella disiens	1/1 (100)	0
Prevotella intermedia	1/1 (100)	0
Prevotella spp	1/1 (100)	0

CE=clinically evaluable; MRSA=methicillin-resistant *Staphylococcus aureus*; MSSA= methicillin-susceptible *Staphylococcus aureus*; SOC=standard of care.

[a]Categories are not mutually exclusive.

[b]One patient was indeterminate due to loss to followup after day 8 visit.

[c]One patient was a failure due to requirement of > 6 weeks of SOC antibiotics.

[d]Two patients were indeterminate due to loss to followup after day 8 visit. Coagulase-negative staphylococci [n/N (%)]: *S. epidermidis*, 6/6 (100); *S. haemolyticus*, 3/4 (75); *S. hominis*, 1/2 (50); *S. pasteuri*, 1/1 (100); *S. simulans*, 1/1 (100).

[e]Coagulase-negative staphylococci [n/N (%)]: *S. epidermidis*, 2/2 (100).

Supplementary Table 3. Baseline Pathogens in Patients with Mixed Infection, with Gram-Positive and Gram-Negative Aerobic Organism in Bone (Safety Population)

Baseline Pathogen, by Patient	Site of Osteomyelitis	Clinical Response at Day 42
**Dalbavancin Group**		
1. MSSA, *Escherichia coli* (plus anaerobes *Bacteroides fragilis, Peptoniphilus harei*)	Hand	Cure
2. *Enterococcus faecalis, Enterobacter cloacae complex* (plus anaerobe *Prevotella intermedia*)	Tibia	Cure
3. MSSA, *Enterococcus faecalis, Escherichia coli, Klebsiella pneumoniae* (plus anaerobes *Bacteroides fragilis, Bacteroides vulgatus*)	Diabetic foot	Cure
4. *Staphylococcus haemolyticus, Streptococcus dysgalactiae, Escherichia coli, Klebsiella pneumoniae*	Foot (not diabetic)	Cure
5. MSSA, *Morganella morganii, Proteus mirabilis* (plus anaerobes *Bacteroides thetaiotaomicron, Porphyromonas asaccharolytica*)	Foot (not diabetic)	Cure
6. MSSA, *Morganella morganii* (plus anaerobe *Finegoldia magna)*	Foot (not diabetic)	Cure
7. *Staphylococcus epidermidis, Enterococcus faecalis, Escherichia hermannii, Pluralibacter gergoviae, Raoultella planticola*	Diabetic Foot	Cure

8. MSSA, *Staphylococcus haemolyticus, Acinetobacter calcoaceticus*	Patella	Cure
9. *Staphylococcus simulans, Pseudomonas aeruginosa*	Tibia	Cure
10. MSSA, *Pseudomonas aeruginosa*	Femur	Cure
11. MSSA, *Enterobacter cloacae complex* (plus anaerobes *Peptoniphilus harei, Prevotella disiens*)	Foot (not diabetic)	Cure
**SOC Group**		
1. MSSA, *Pseudomonas aeruginosa, Raoultella planticola, Serratia marcescens*	Tibia	Failure
2. MRSA, *Klebsiella pneumoniae, Proteus mirabilis*	Femur	Cure

MRSA=methicillin-resistant *Staphylococcus aureus*; MSSA= methicillin-susceptible *Staphylococcus aureus*; SOC=standard of care.

Supplementary Figure 2. CRP levels in the mITT population in the A) dalbavancin group and B) SOC group. CRP=C-reactive protein; mITT=modified intent-to-treat; SOC=standard of care.

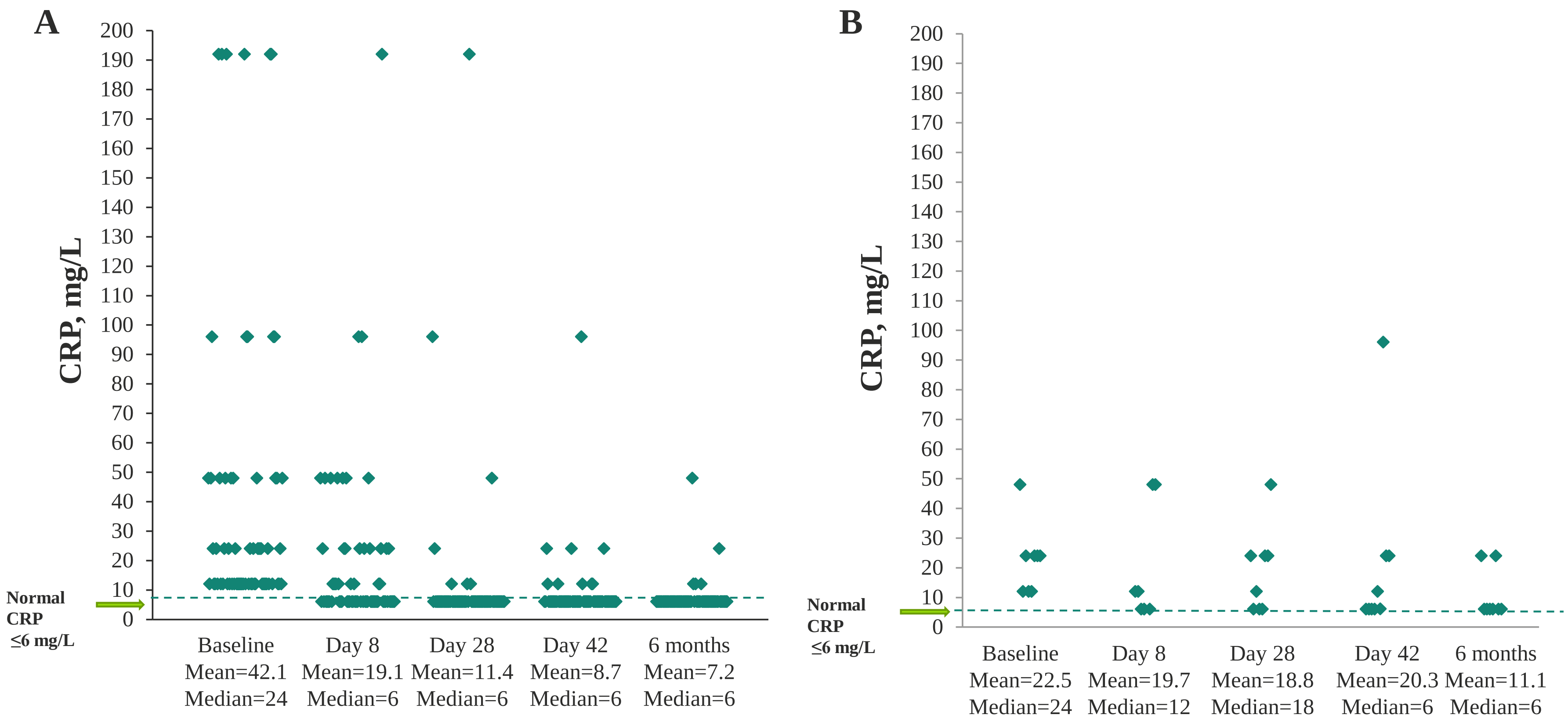

Supplement: ofy331_suppl_supplementary_material [file ofy331_suppl_supplementary_material.pdf]
